# Supplementary material for: Understanding the Significance of Biochemistry in the Storage, Handling, Purification, and Sampling of Amphiphilic Mycolactone
Source: Toxins (Basel). 2019 Apr 4;11(4):202. doi: 10.3390/toxins11040202 (PMC6520765; doi:10.3390/toxins11040202)

HPLC-grade Acetonitrile Blank

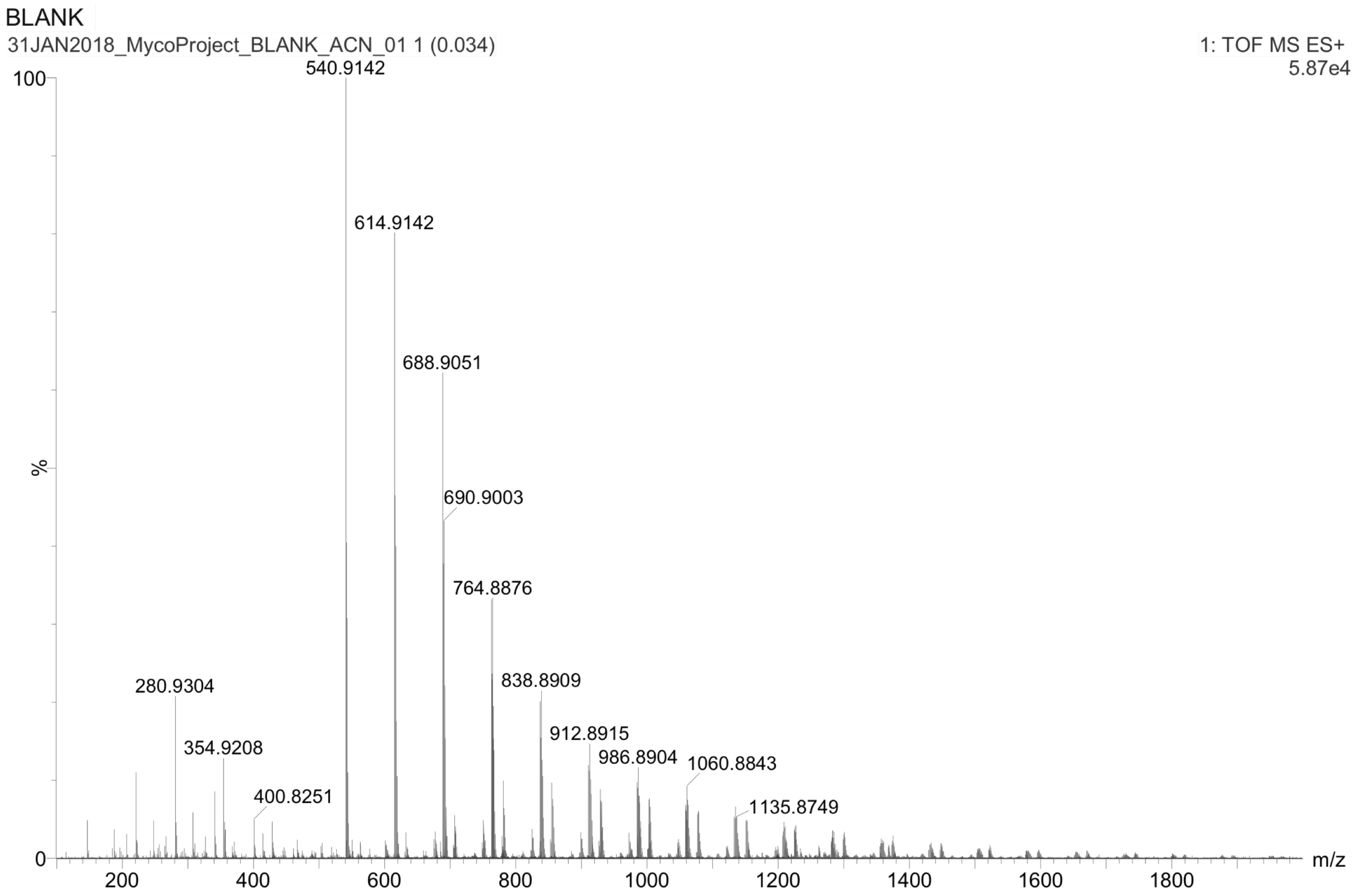

Sample #1
TLC Purified

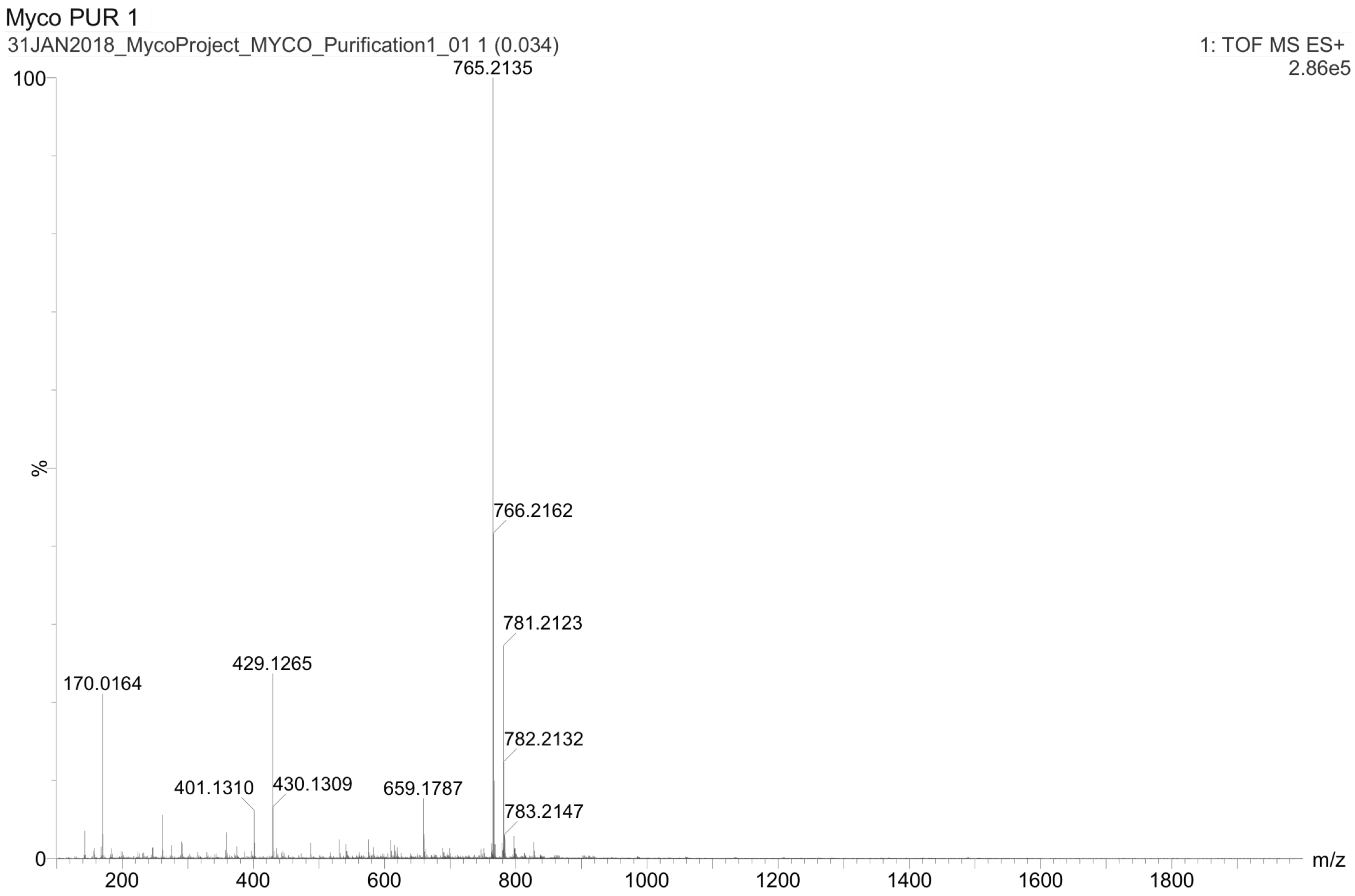

Sample #3
TLC Purified

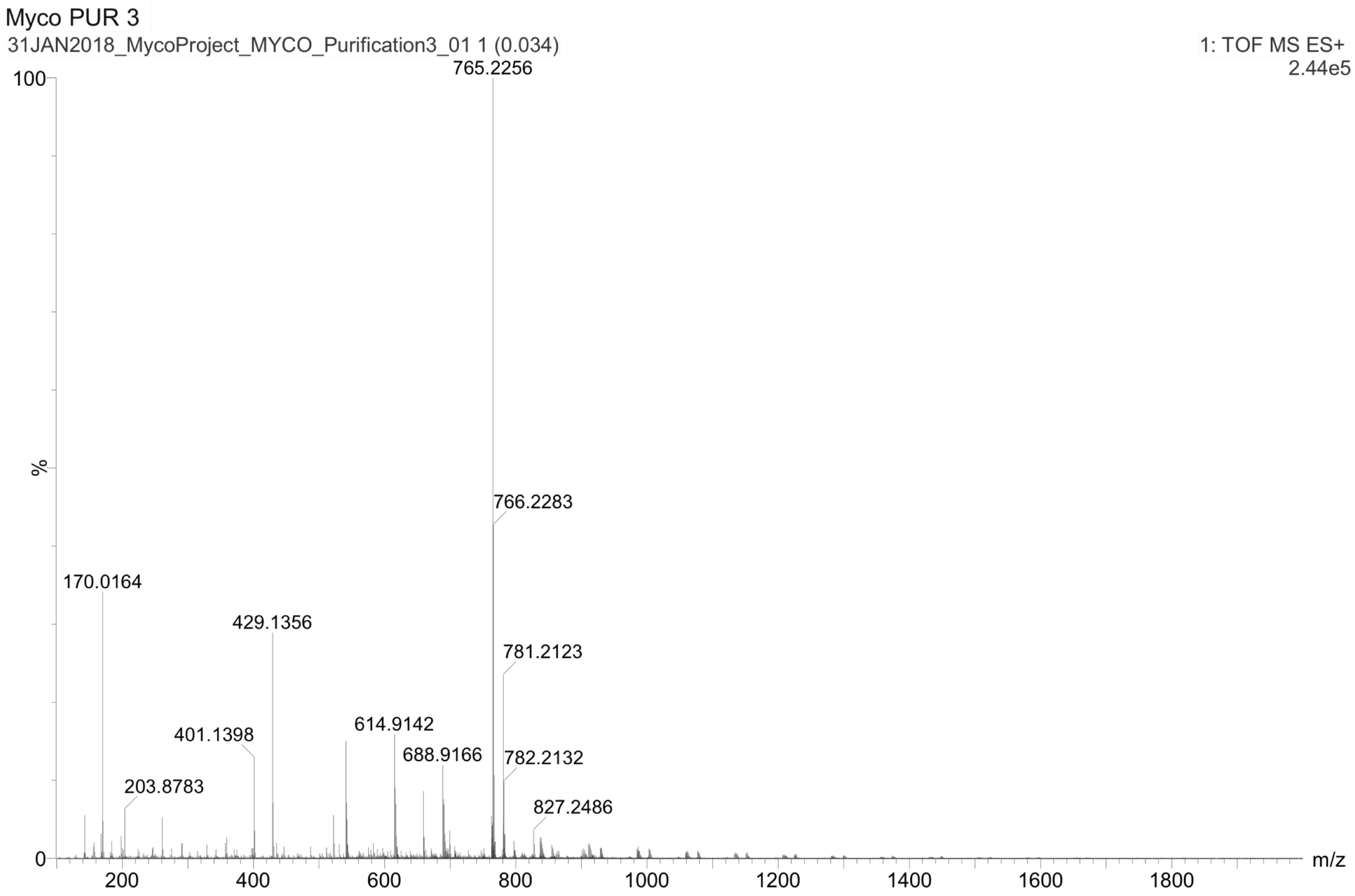

Sample #5
Ethanol Extract

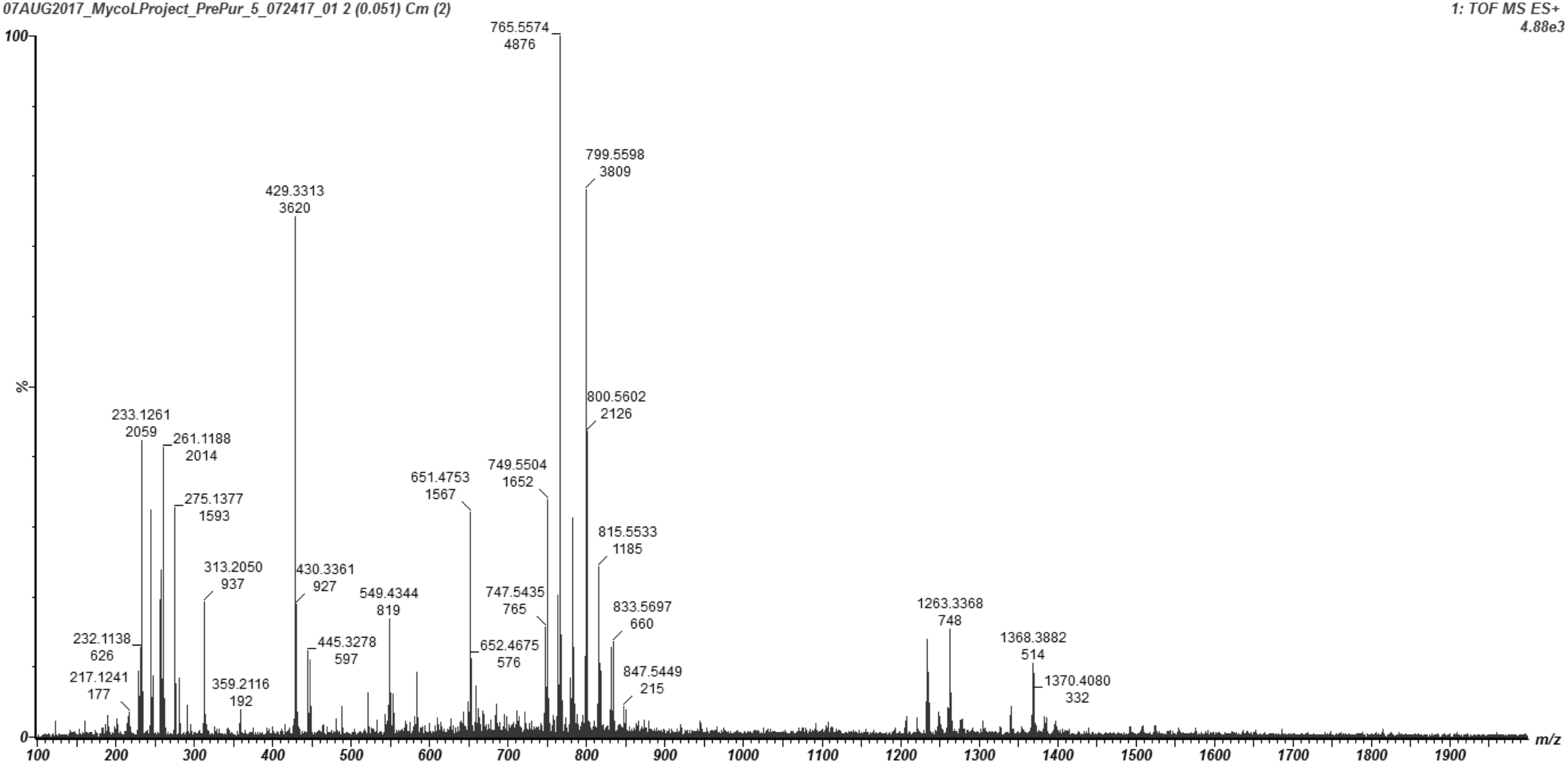

Sample #5
TLC Purified

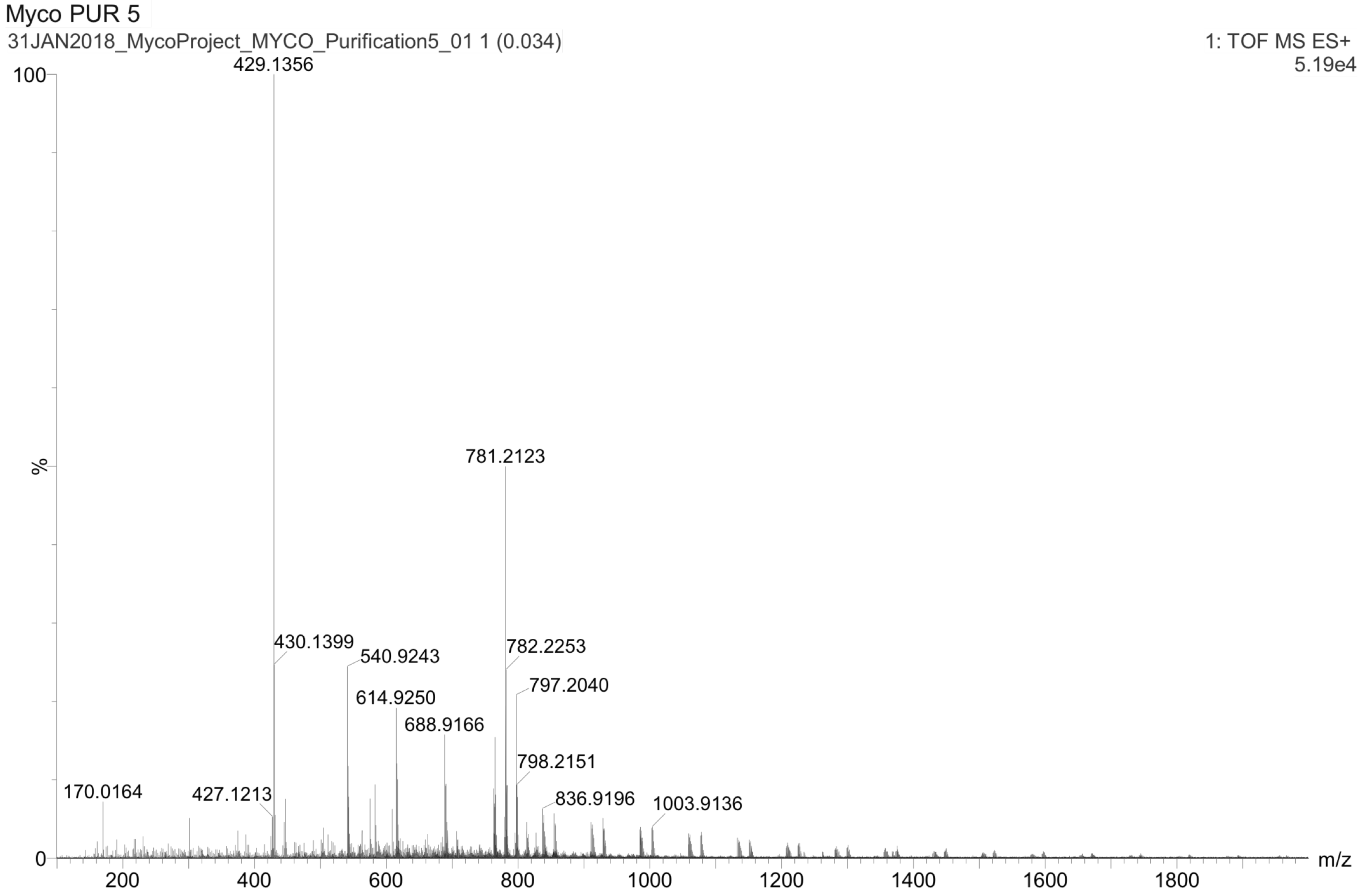

Sample #6
Ethanol Extract

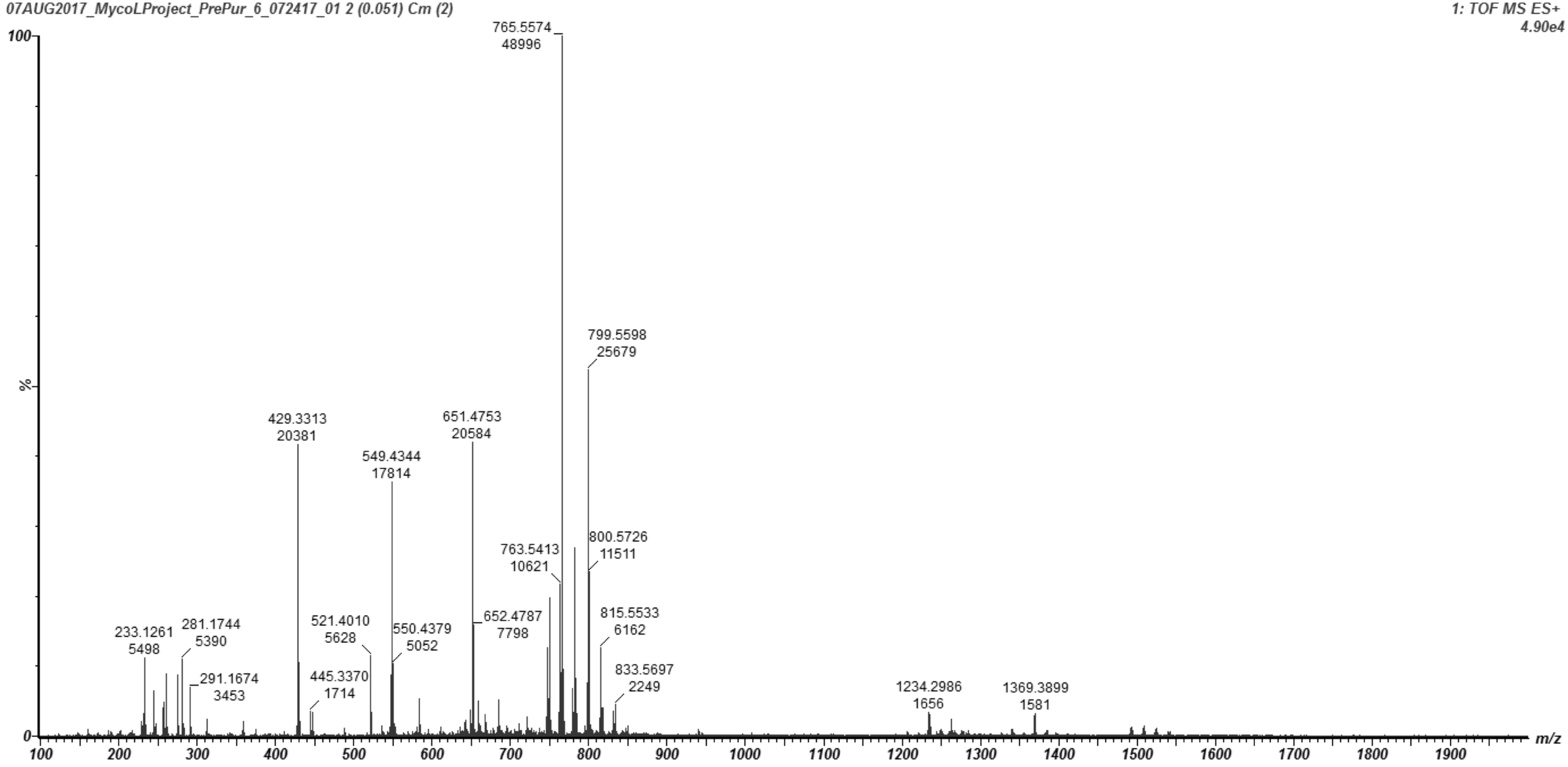

Sample #6
TLC Purified

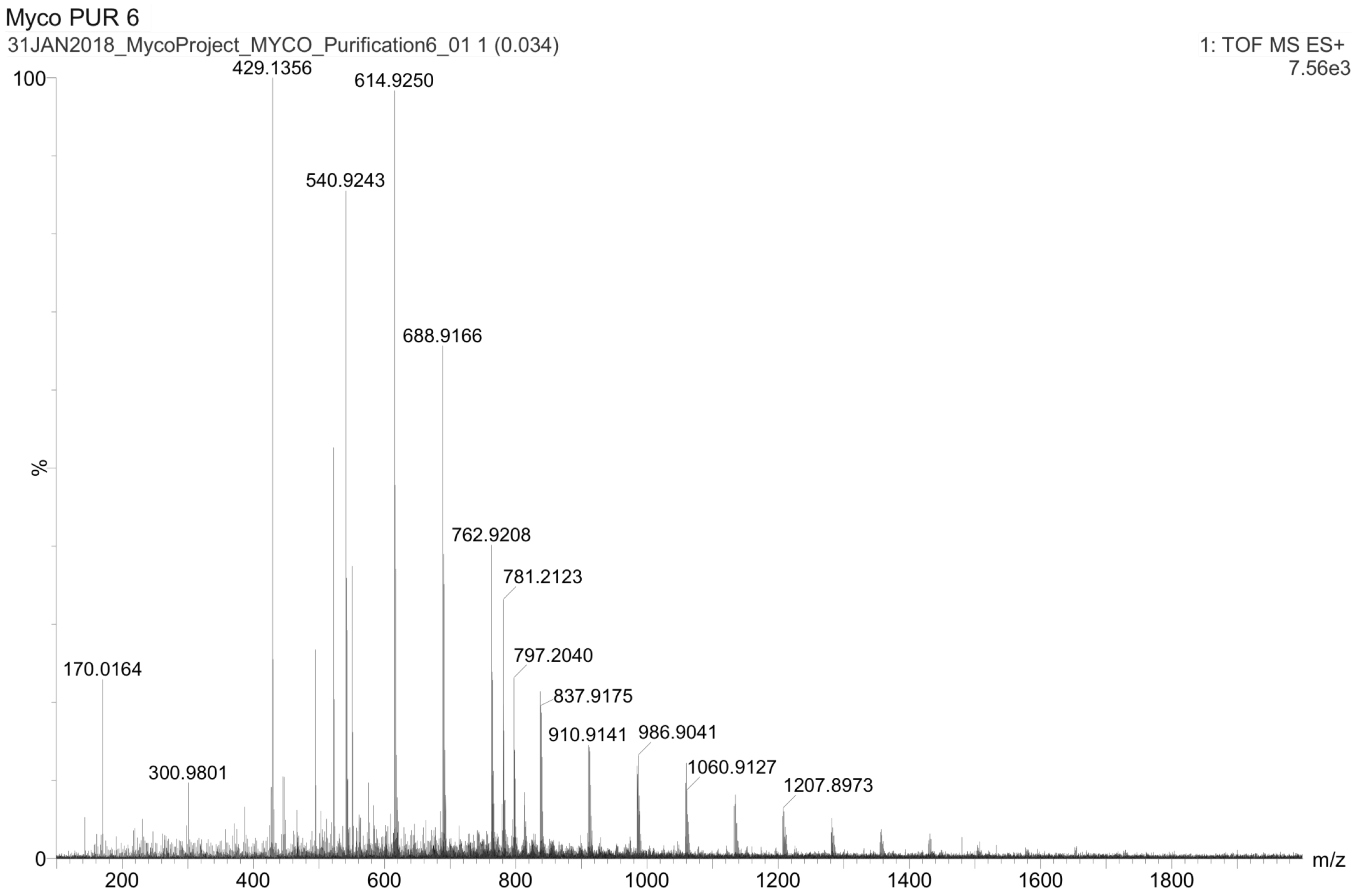

Sample #7
Ethanol Extract

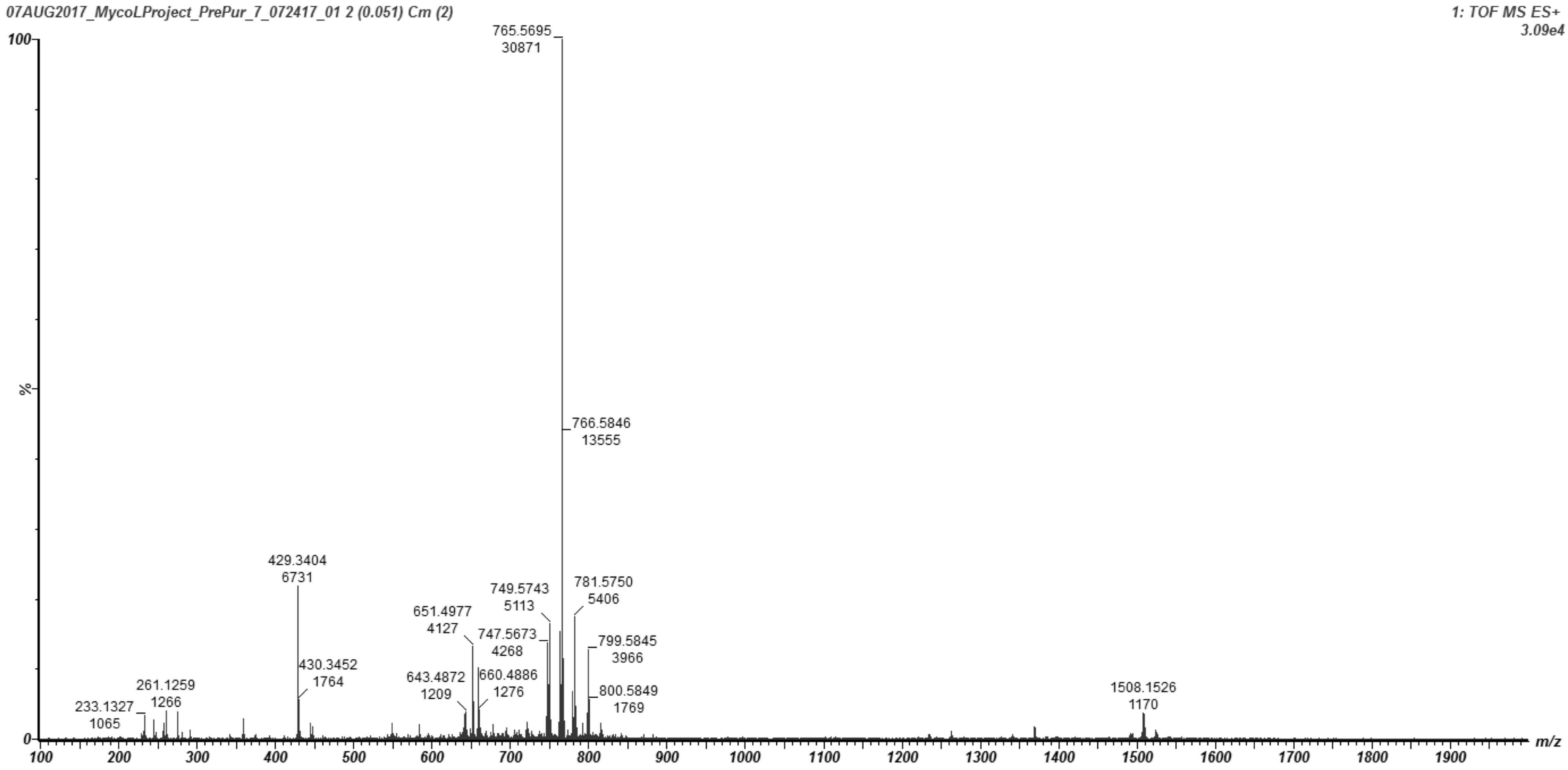

Sample #7
TLC Purified

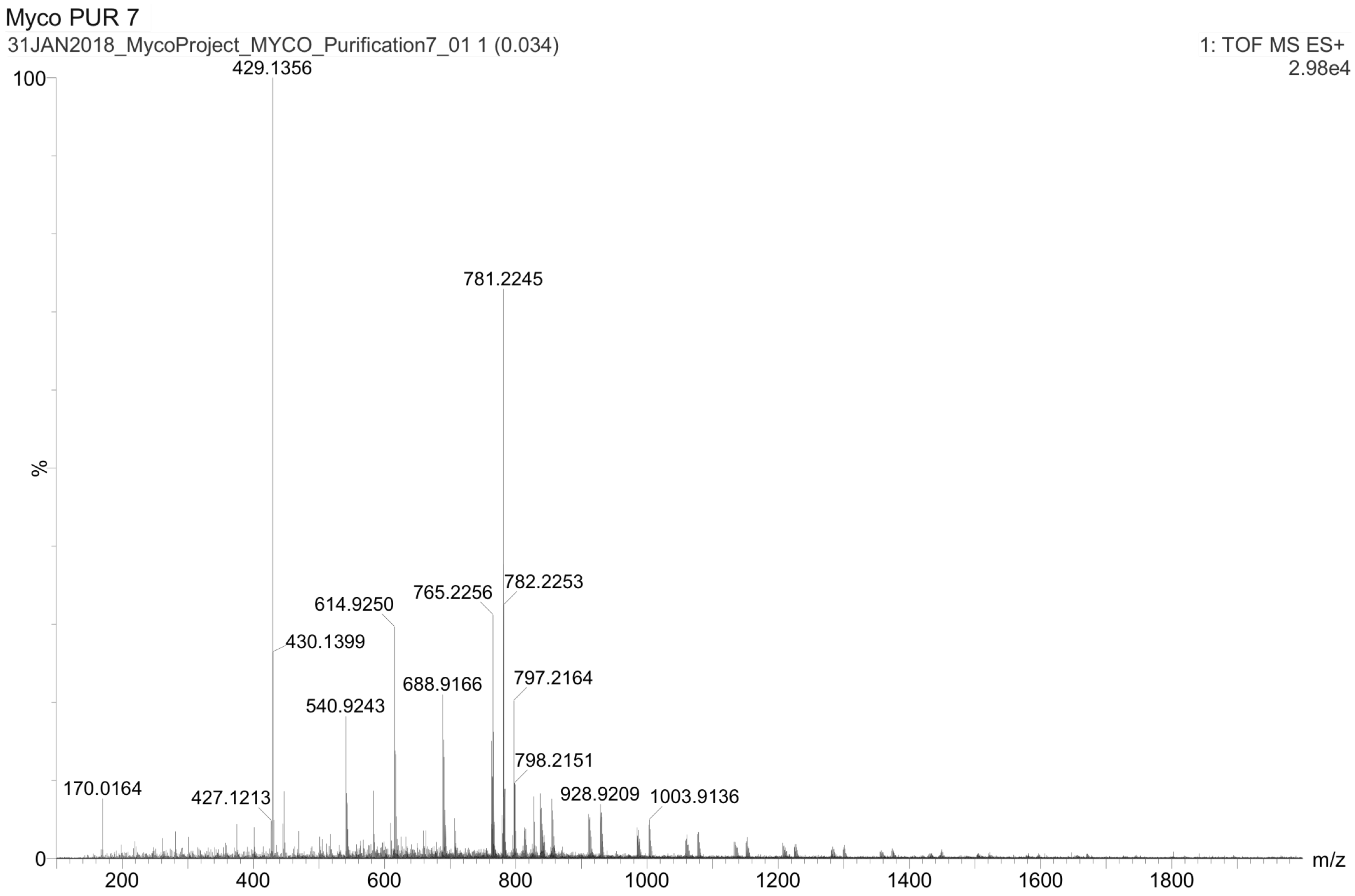

Sample #8
Ethanol Extract

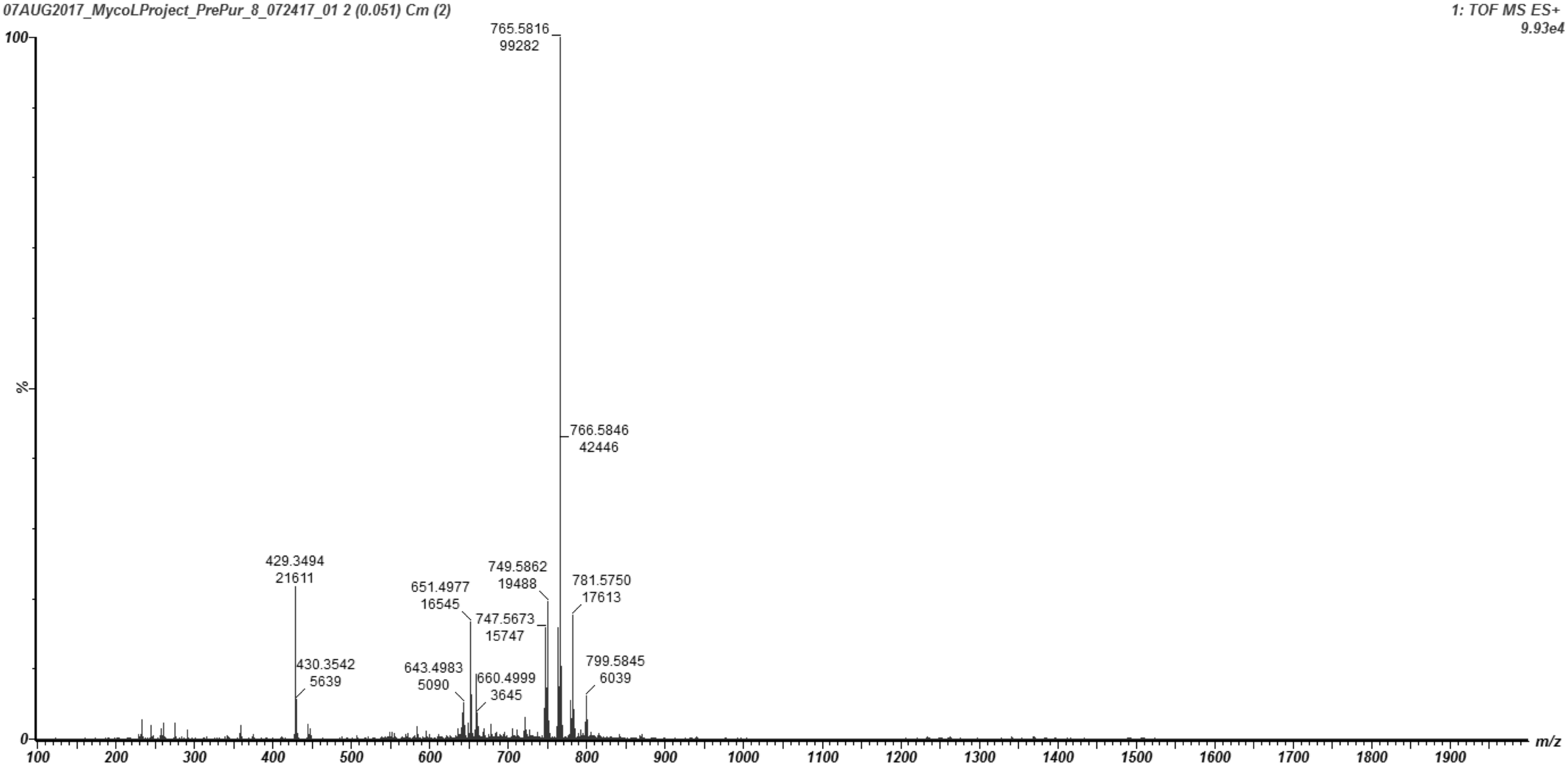

Sample #8
TLC Purified

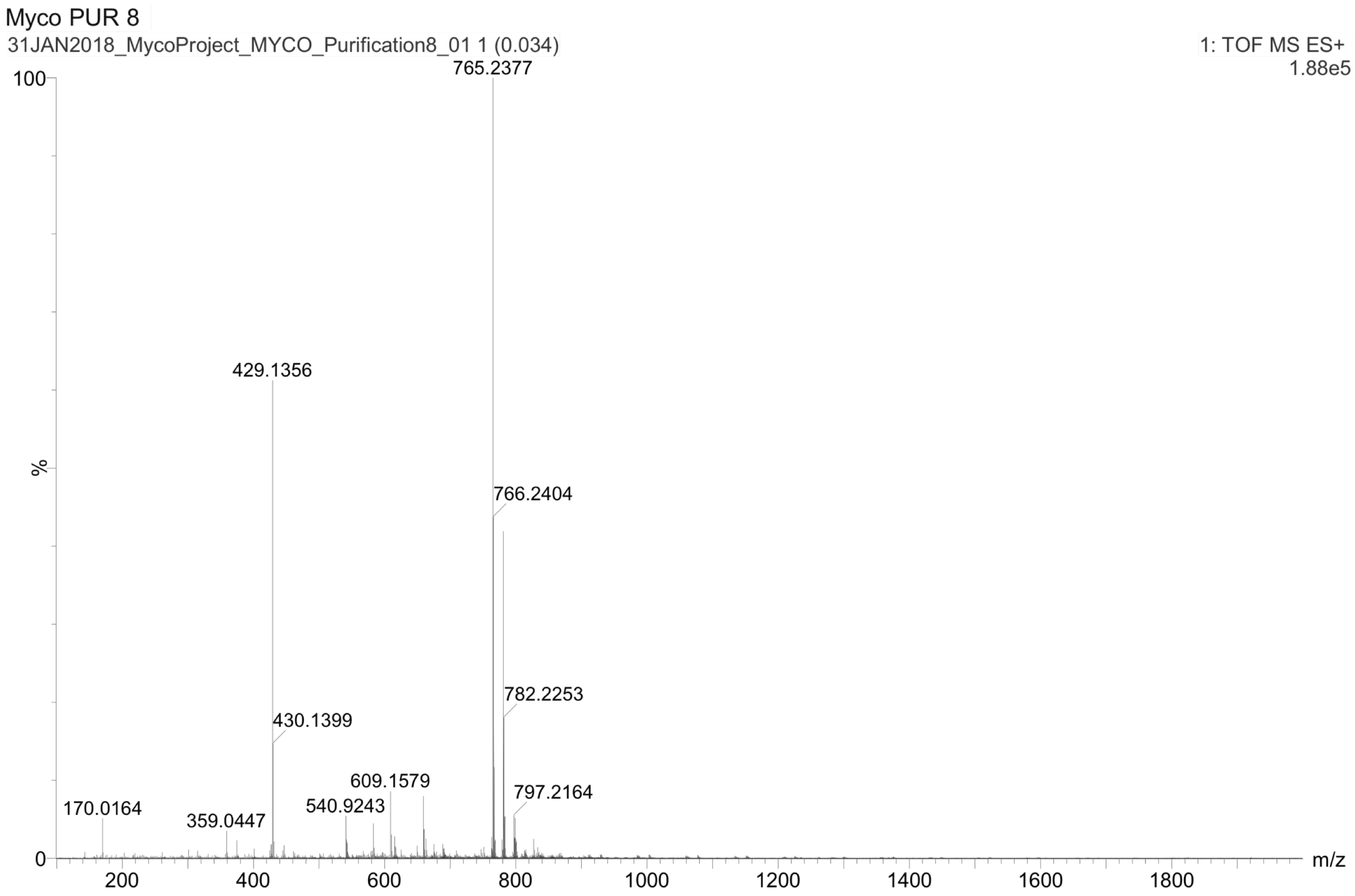

Sample #9
Ethanol Extract

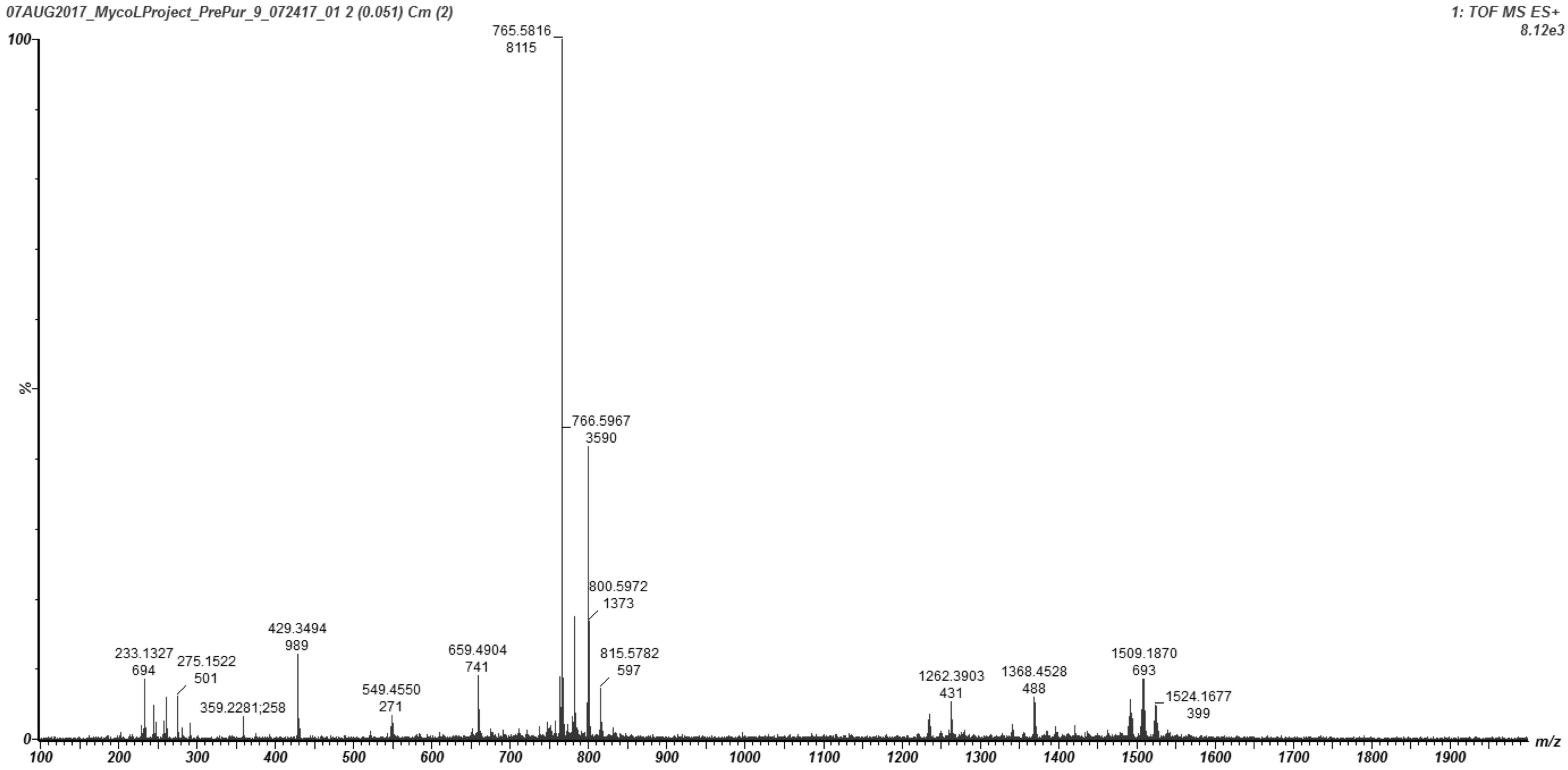

Sample #9
TLC Purified

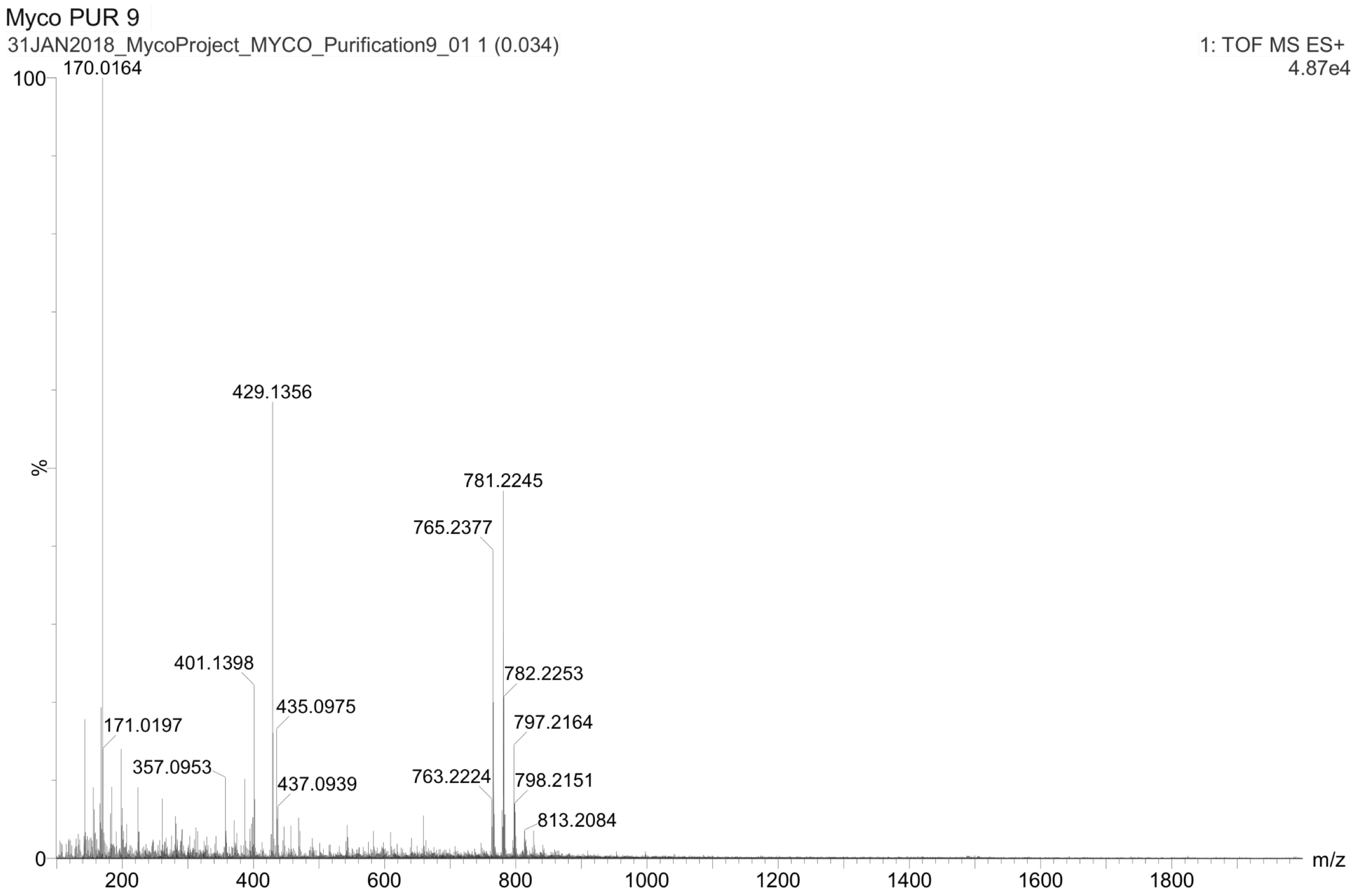

Sample #10
Ethanol Extract

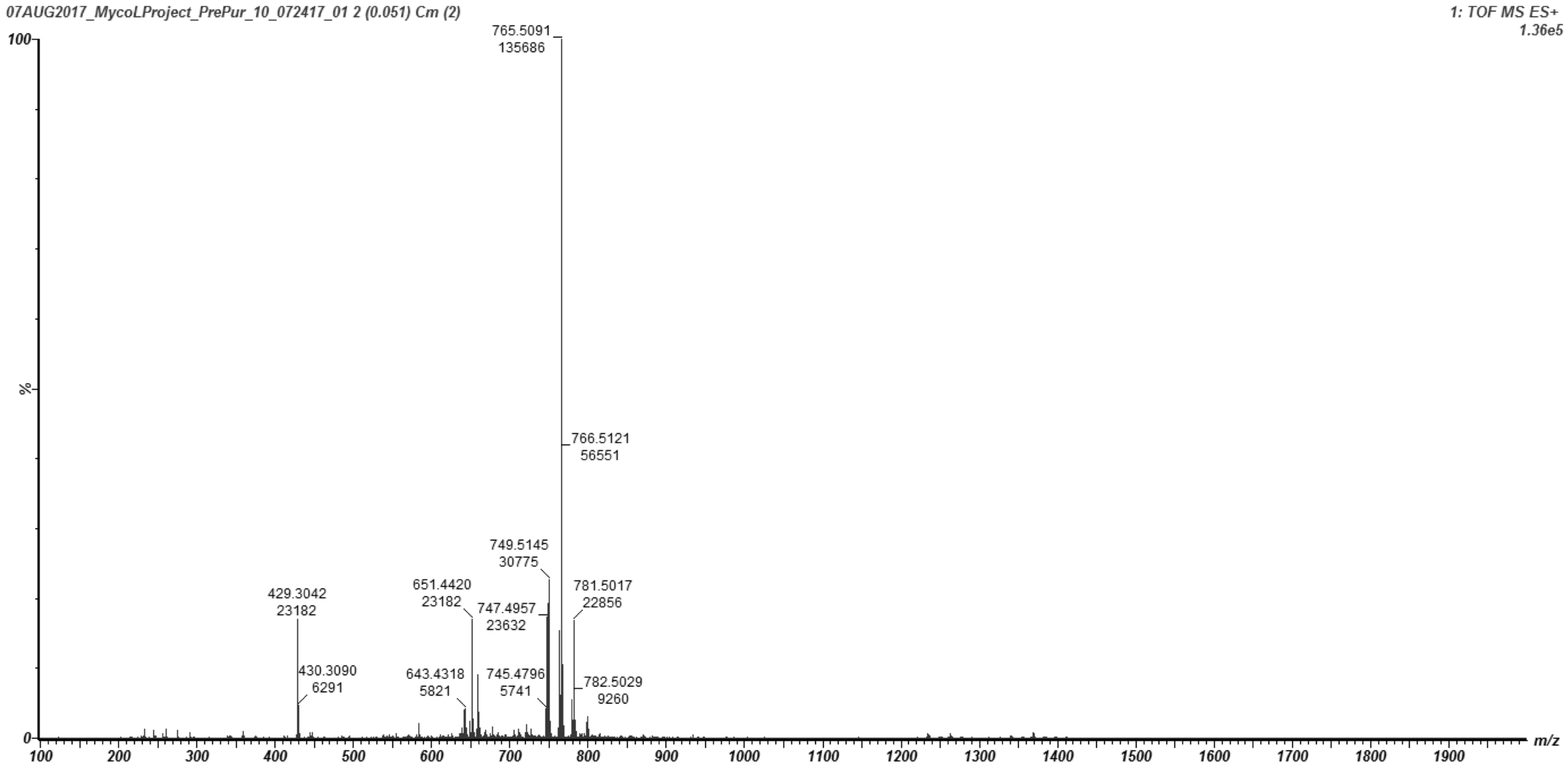

Sample #10
TLC Purified

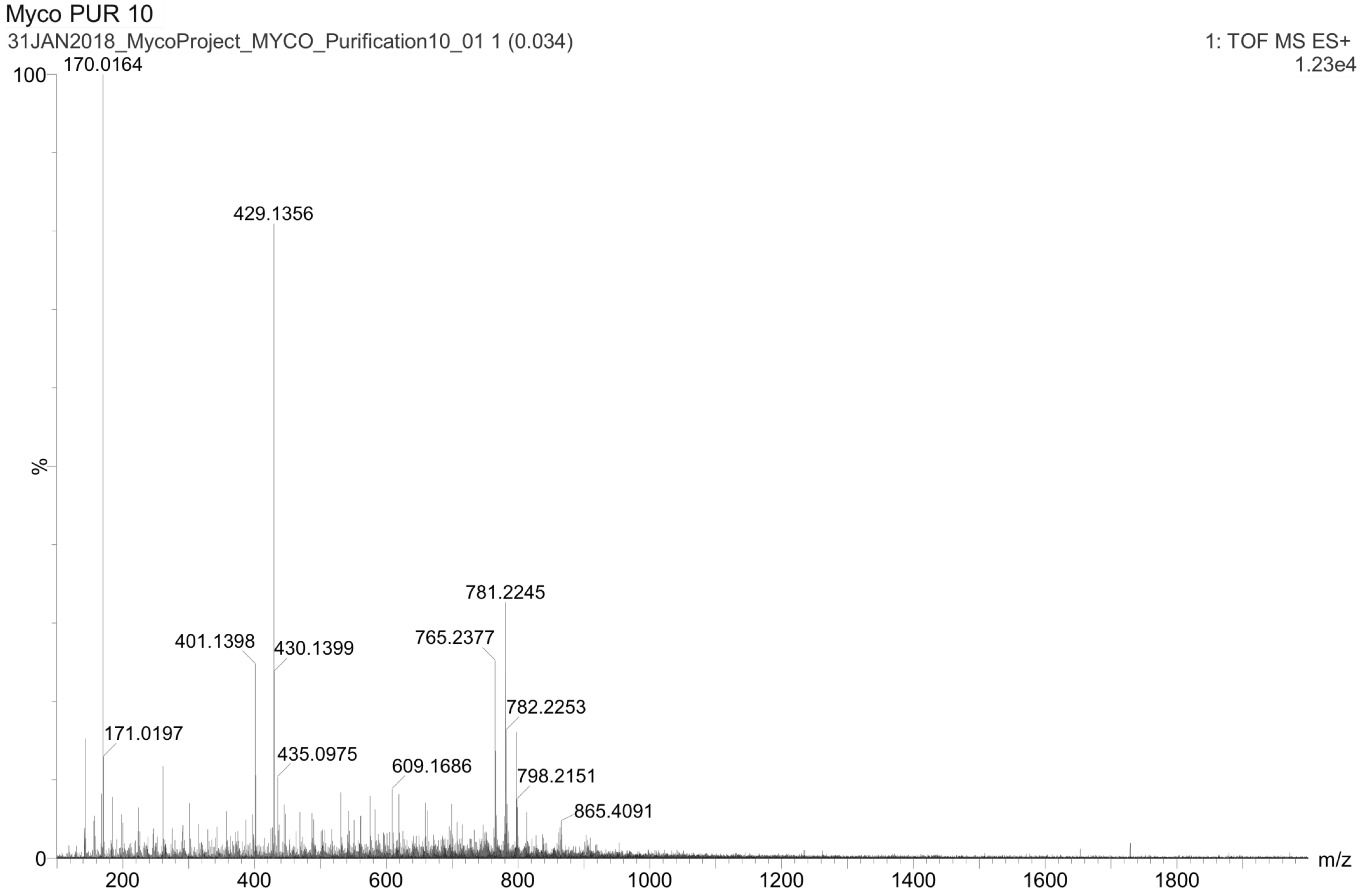

Sample #11
Ethanol Extract

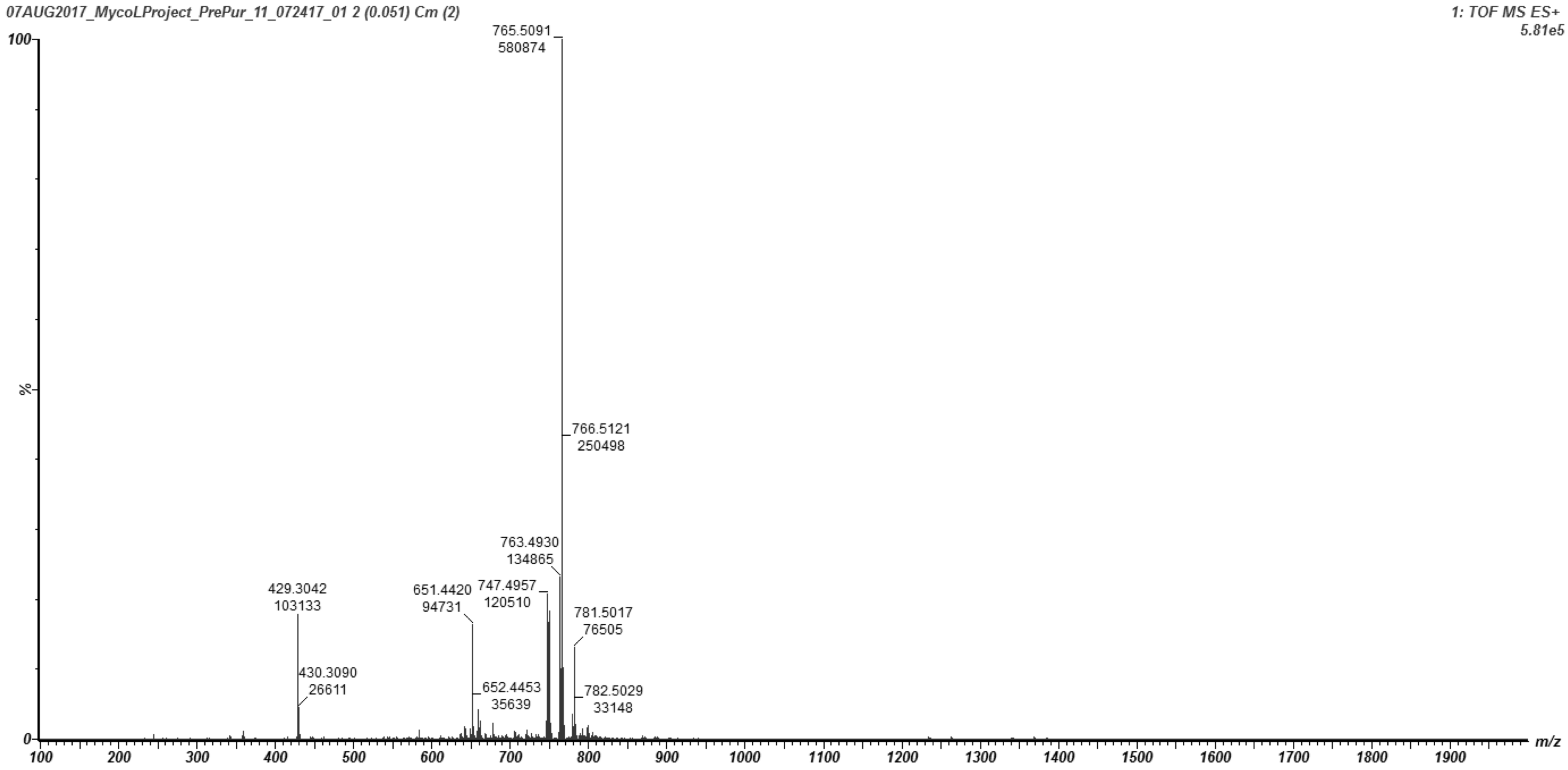

Sample #11
TLC Purified

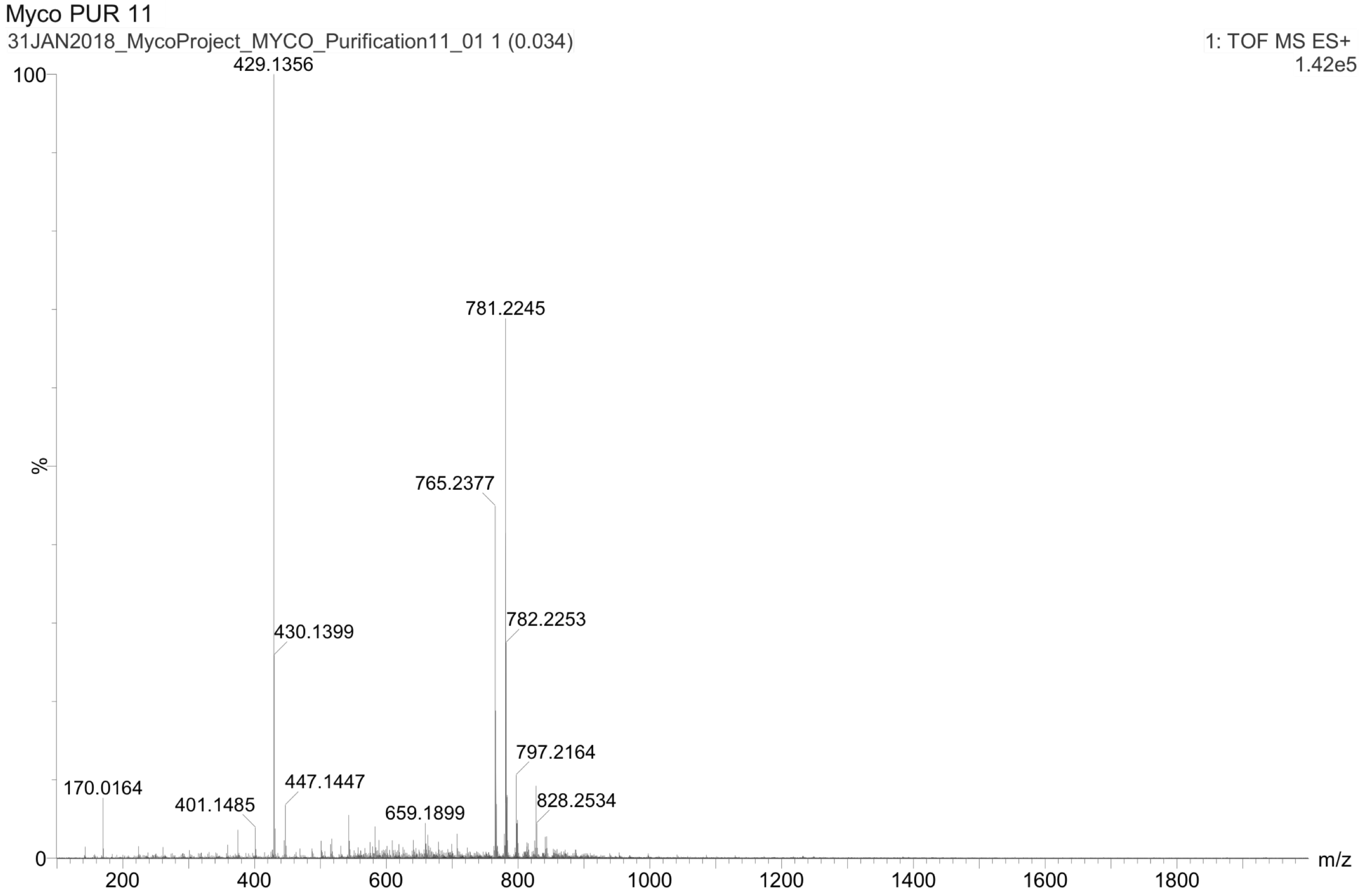

Supplement: Supplementary file 1 [file toxins-11-00202-s001.pdf]
